# Demographic Features of Cleveland Alzheimer's Disease Research Center Participants Who Were Initially Undecided or Change Their Mind Regarding Brain Donation

**DOI:** 10.1002/alz70858_100963

**Published:** 2025-12-25

**Authors:** David Silva, Lyndsi Powell, Alexsandra Kovacevich, Jagan A. Pillai, Audrey Lynn, Sunah Song, Brian S Appleby

**Affiliations:** ^1^ Case Western Reserve University, Cleveland, OH, USA; ^2^ University Hospitals Cleveland Medical Center, Cleveland, OH, USA; ^3^ University Hospitals Cleveland Medicine Center, Cleveland, OH, USA; ^4^ Cleveland Clinic, Cleveland, OH, USA; ^5^ Cleveland Clinic Lou Ruvo Center for Brain Health, Las Vegas, NV, USA; ^6^ Department of Population and Quantitative Health Sciences, Case Western Reserve University School of Medicine, Cleveland, OH, USA; ^7^ Department of Population and Quantitative Health Sciences, Case Western Reserve University, Cleveland, OH, USA; ^8^ Cleveland Institute for Computational Biology, Case Western Reserve University, Cleveland, OH, USA; ^9^ Case Western Reserve University School of Medicine, Cleveland, OH, USA

## Abstract

**Background:**

Brain donation from participants enrolled in nationwide Alzheimer's Disease Research Centers (ADRCs) provide a valuable resource for ADRD researchers. Prior research has focused on factors associated with brain donation that include cognitive status, age, race/ethnicity, cultural/religious beliefs, and psychosocial factors. This previous research has focused primarily on whether or not participants enroll in brain donation programs, but has not examined factors associated with participants that are undecided and/or change their donation status. Our study focuses on demographic features of participants in the Cleveland ADRC (CADRC) who are undecided regarding brain donation or who changed their donation status over time.

**Method:**

The CADRC database was queried to examine demographic variables related to participants’ brain donation status. Three brain donation statuses were used for these analyses: yes, no, and undecided. Analyses were performed to examine differences between brain donation status at the initial visit and for participants that changed their brain donation status over time.

**Result:**

Data from 277 CADRC participants were included in the analyses. At the initial visit, participants that were undecided regarding brain donation were associated with female gender (*p* = 0.025), younger age (*p* = 0.017), not married/partnered (*p* = 0.035), and had a study partner that was not a relative (*p* = 0.008). Participants that changed their brain donation status over time were associated with white race (*p* = 0.01), older age at enrollment (*p* = 0.04), and had more CADRC visits (*p* <0.001). Participants with lower education were associated with changing their status from undecided to yes (*p* = 0.02). At one of the clinical enrollment sites, participants who were consented by one specific staff member were more likely to enroll in the brain donation program compared to all other staff members combined at that enrollment site, both at the initial visit (*p* = 0.012) and throughout the study (*p* = 0.001).

**Conclusion:**

This preliminary study of CADRC participants demonstrates important demographic differences in those that are undecided regarding brain donation and in those who change their status over time. Similar to other studies, interactions with study staff also affects brain donation enrollment. Future studies should examine individuals who are undecided regarding brain donation as well as examine longitudinal changes in brain donation status.

